# Supplementary material for: The GJB2 (Cx26) Gene Variants in Patients with Hearing Impairment in the Baikal Lake Region (Russia)
Source: Genes (Basel). 2023 Apr 28;14(5):1001. doi: 10.3390/genes14051001 (PMC10218609; doi:10.3390/genes14051001)
Supplement: Supplementary file 1 [file genes-14-01001-s001.zip › genes-2334613-supplementary.pdf]

Supplementary materials

**Chapter S1**	Sanger sequencing of identified 14 variants of the *GJB2* gene in 165 patients with HI in the Republic of Buryatia	2
**Chapter S2**	Pathogenicity analysis *in silico* of c.-254C>T and c.-49G>A variants of the *GJB2* gene	6
**Chapter S3**	Haplotype analysis of the c.-23+1G>A, c.35delG and c.235delC pathogenic variants	7

Chapter S1. Sanger sequencing of identified 14 variants of the *GJB2* gene in 165 patients with HI in the Republic of Buryatia

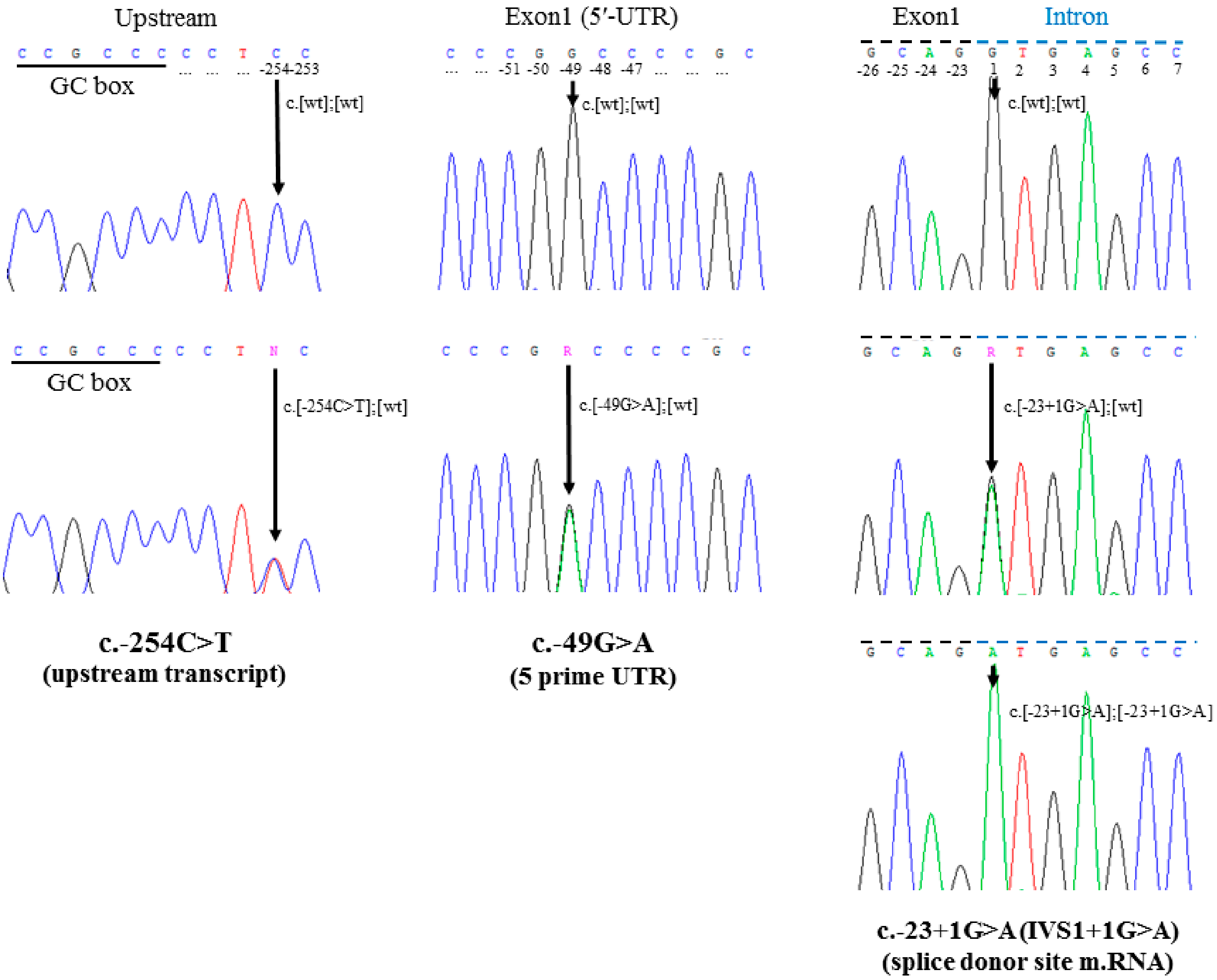

Figure S1. The variants identified in the non-coding regions of the *GJB2* gene.

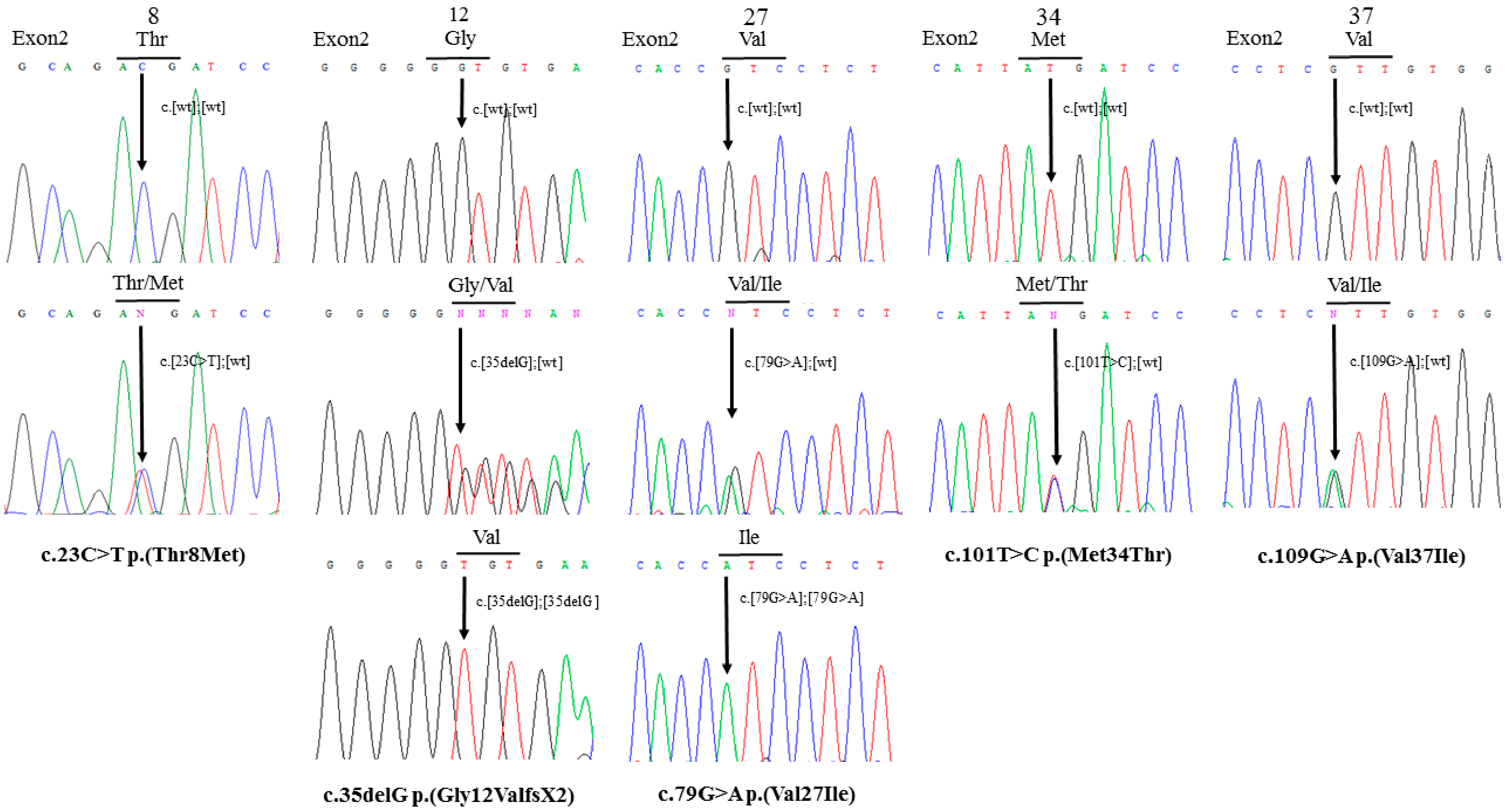

Figure S2. The variants identified in the coding region (exon 2) of the *GJB2* gene.

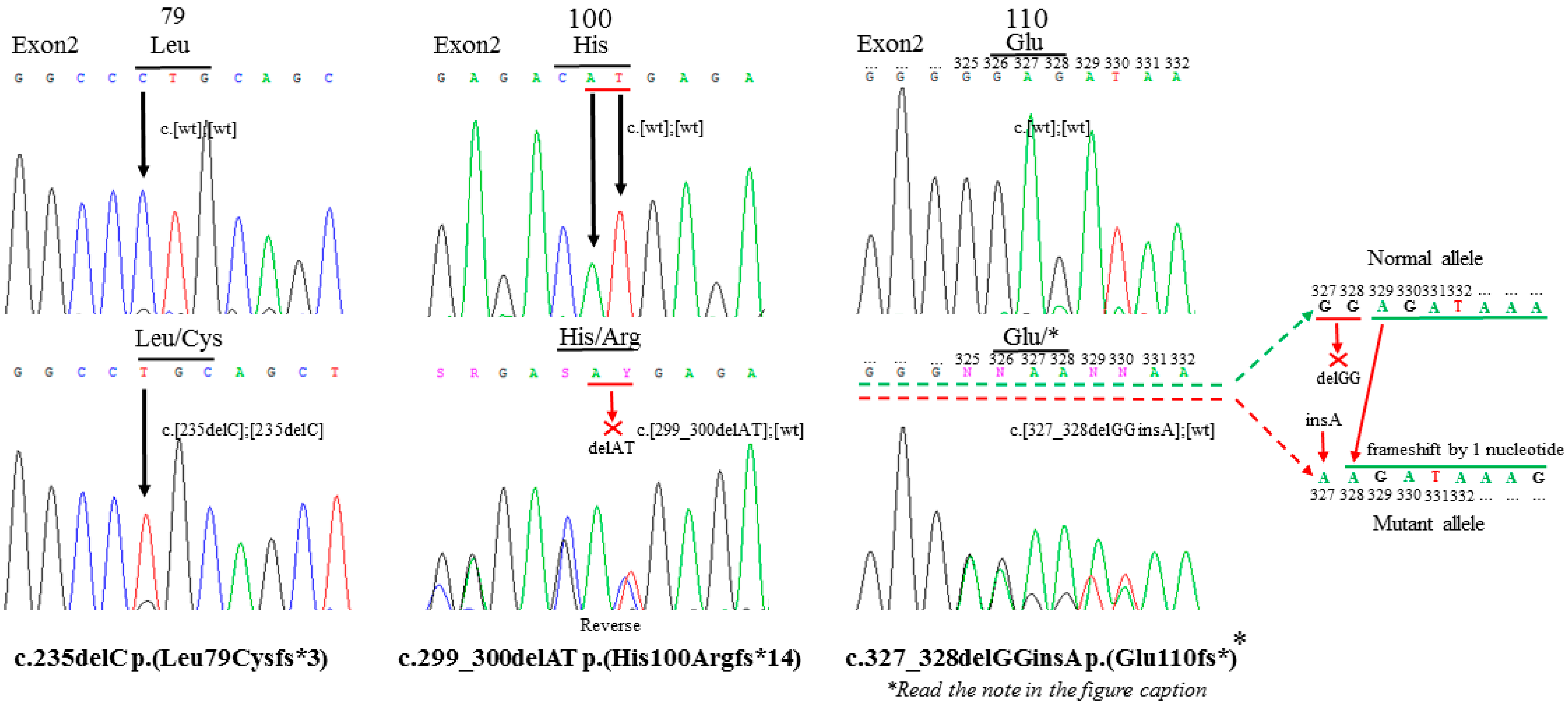

Figure S2 (continued). The variants identified in the coding region (exon 2) of the *GJB2* gene.

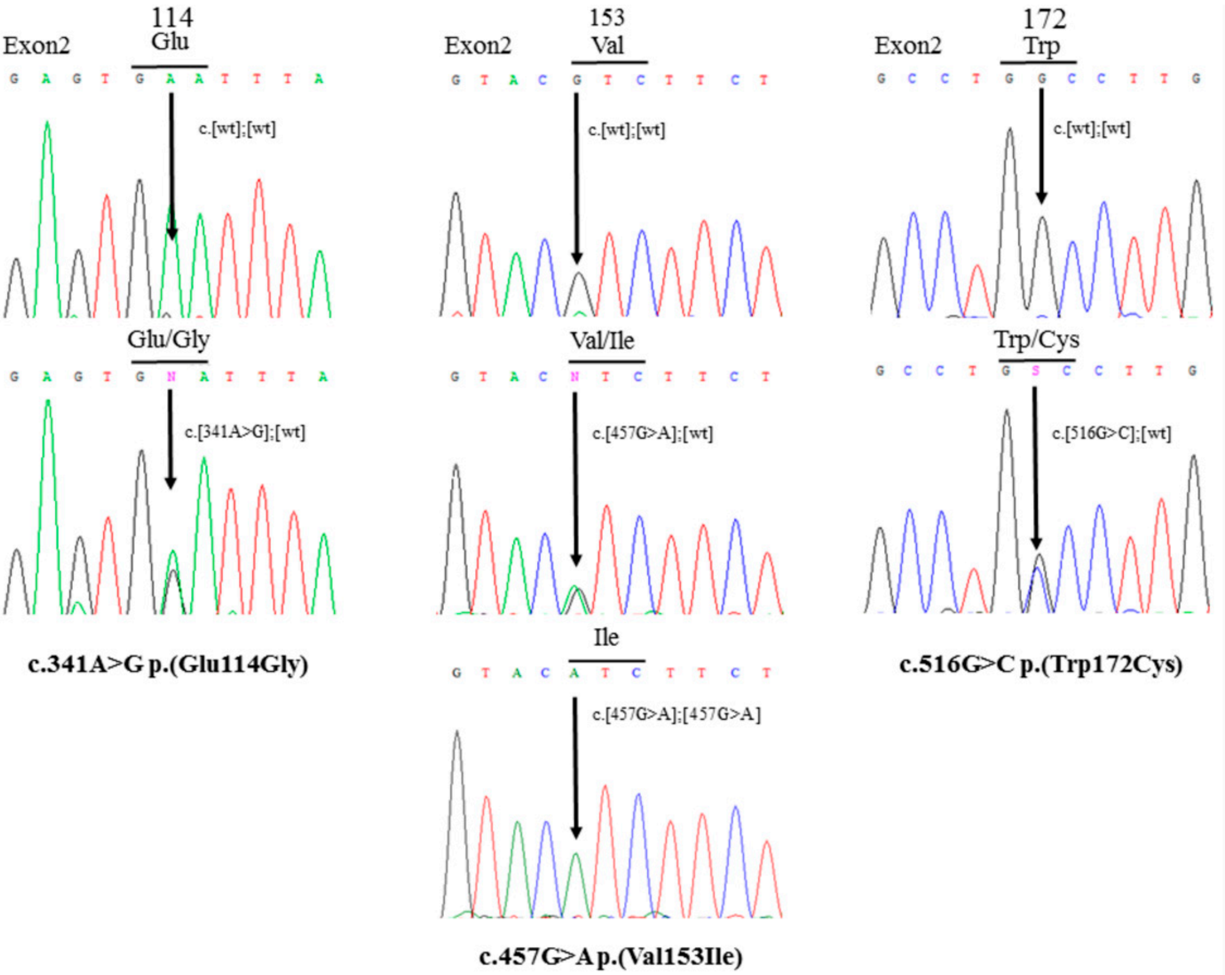

Figure S2 (end). The variants identified in the coding region (exon 2) of the *GJB2* gene.

Chapter S2. Pathogenicity analysis *in silico* of c.-254C>T and c.-49G>A variants of the *GJB2* gene

The c.-49G>A variant of the *GJB2* gene is not currently announced in known databases of annotated variants such as HGMD, ClinVar, DVD. The c.-254C>T (rs1411911768) variant was annotated in Franklin database (https://franklin.genoox.com/clinical-db/variant/snp/chr13-20767153-G-A?app=assessment-tools) and classified as a variant with uncertain significance. To predict effect on the secondary structure of mRNA of variant c.-254C>T/U (critical regulatory region consisting of 29 bp region) and variant c.-49G>A (21 bp, from -59 to 39), we used the Sfold software (https://sfold.wadsworth.org/cgi-bin/srna.pl) (Figure S3).

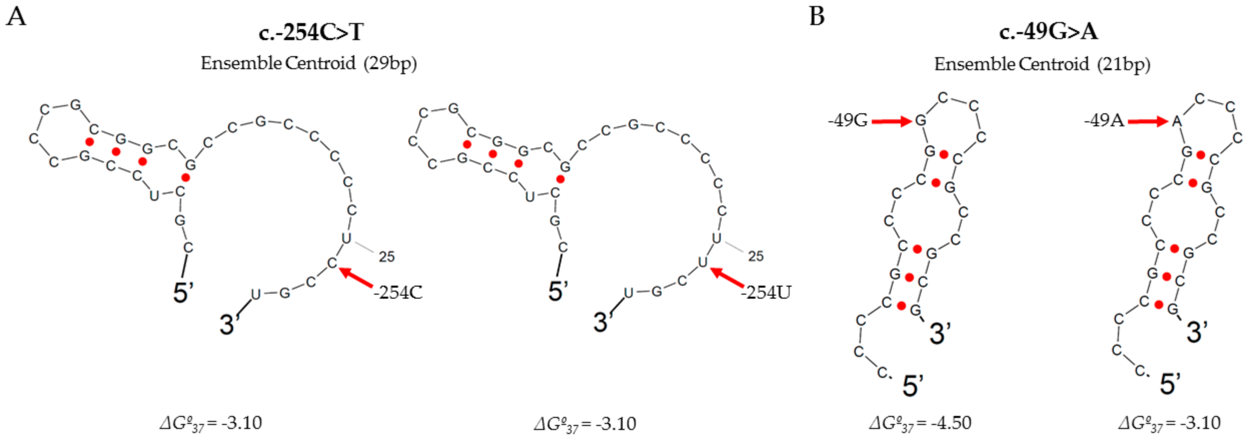

Figure S3. Ensemble centroid structure diagram for two variants c.-254C>T and c.-49G>A of the *GJB2* gene, generated in the Sfold software (https://sfold.wadsworth.org/cgi-bin/srna.pl). **A** - The centroid structure of the ensemble consisting of 29 bp promoter region of *GJB2* gene in which two GC-boxes and the c.-254C>T variant are located. On the left is the normal -254 region, on the right is the mutated -254T/U region. **B** - The centroid structure of the-ensemble consisting of 21 bp noncoding exon 1 of the *GJB2* gene, in which the c.-49G>A variant is located. On the left is the normal -49 region, on the right is the mutated region -49A.

In general, based on the location of the noncoding c.-254C>T and c.-49G>A substitutions in the sequence of the proximal promoter and noncoding regions of the *GJB2* gene and the *in silico* analysis of their possible effect on the posttranscriptional regulation of *GJB2* gene expression, we suggest that these variants do not have a pathogenic effect, but, most likely, are benign, with no clinical significance.

Chapter S3. Haplotype analysis of the c.-23+1G>A, c.35delG and c.235delC pathogenic variants in the *GJB2* gene.

Table S1. Detection and primer sequences of 12 SNP-markers, flanking the region of 84536 kb, around the *GJB2* gene in chromosome 13.

#	SNP-markers	Nucleotide Change	Position	Forward primers	Reverse primers	PCR-product (bp)	Restriction/Sanger sequences
1	rs1932429	C>A	20197576	F5′-GCA-TTA-CTT-ATC-CTT-GCT-AAT-3′	R5′-GTG-TTC-TCT-GAC-TTA-GTC-TCA-3′	294	*Taq*I
2	rs5030701	G>A	20195087	F5′-TTC-CCT-CAC-CCT-TTT-CCC-AC-3′	R5′ – CGG-AGT-GGC-TGT-GAT-AAT-ACG-3′	253	*Mbo*II
3	rs7987144	T>C	20194005	F5′-CCT-CAT-CCG-CTT-GCG-CTG-TGC-A-3′	R5′-CAA-GCC-CCT-TCC-TGC-TCG-ATG-C-3′	229	*Alu*I
4	rs7994748	T>C	20191991	F5′-AGG-ACC-CTT-GTT-CGC-GAA-GAG-3′	R5′-TTC-TCA-CCA-GTG-TCT-GAA-ATT-3′	265	*Msp20*I
5	rs2274084	G>A	20189503	F5′-TCT-TTT-CCA-GAG-CAA-ACC-GC-3′	R5′-TCT-AAC-AAC-TGG-GCA-ATG-C-3′	733	Sanger sequences
6	rs2274083	A>G	20189241				
7	rs3751385	T>C	20188817	F5′-TCT-GGA-ATT-TGC-ATC-CTG-CTG-3′	R5′-CCT-AAC-AGC-CTG-GGG-TCT-C-3′	359	*Bmt*I
8	rs5030700	C>T	20187970	F: 5′-GCA-CCT-AAC-AAC-ATT-GTA-GCC-3′	R: 5′-TTT-AAC-GAC-AGA-AAC-TTC-TCC-C-3′	310	*SfaN*I
9	rs11841024	A>G	20186219	F5′-CCA-ACA-CTT-TGG-GAG-GCT-CAC-3′	R5′-TTA-AAA-GTT-CTG-TGT-TTT-TTG-ATG-TTG-TTG-TAG-3′	290	*Alu*I
10	rs2313477	T>C	20184184	F5′-CAG-CCC-CAC-ATT-CCT-TCA-C-3′	R5′-TGT-TAA-AAA-GCT-AAG-TCA-CTT-GAG-CCA-TAA-CGT-3′	285	*AccB7*I
11	rs747931	T>C	20126789	F5′-AAA-GGC-GGG-TCG-GCA-CCC-CTA-3′	R5′-CAC-TGT-GCC-TGG-CCC-CAG-GAA-3′	327	*BslF*I
12	rs2031282	C>T	20113040	F5′- CTG-AGT-ACC-AAT-CTC-ACC-AC-3′	R5′- TGC-ACA-GGG-ATA-ATA-ACC-GC-3′	230	*Bst*VI

Note. Positions of SNP markers were defined according to GRCh38.p13 Genome Assembly (https://www.ncbi.nlm.nih.gov/assembl).

Table S2. Reconstruction of the *GJB2*-haplotypes in patients homozygous for c.-23+1G>A, c.35delG or c.235delC pathogenic *GJB2* variants.

**c.-23+1G>A-haplotypes (1-3-5-6-9-10)**	**Yakuts** [39] (n=222)	**Tuvinians** [39] (n=12)	**Evenks** [39] (n=2)	**Russians** [39] (n=2)	**Mongolians** [14] (n=8)	***Turks*** [14] (n=6)	**Buryats** [This study] (n=2)	**Total** (n=254)
**C C G A A C**	**99.5% (221)**	**100% (12)**	**100% (2)**	**100% (2)**	**87.5% (7)**	**100% (6)**	**100% (2)**	**99.2%**
A C G A A C	0.5% (1)	0	0	0	0	0	0	0.4%
C C A A A C	0	0	0	0	12.5% (1)	0	0	0.4%

**c.35delG-haplotypes (7-8-11-12)**	**Belgians** [6] (n=70)	**British** [6] (n=60)	**Americans** [6] (n=98)	**Russians** [This study] (n=28)	**Total** (n=256)
**T C T C**	**58.6% (41)**	**58.3% (35)**	**63.3% (62)**	**64.3% (18)**	**60.9%**
T C C C	27.14% (19)	26.7% (16)	20.4% (20)	17.9% (5)	23.4%
T C T T	10.0% (7)	8.3% (5)	11.2% (11)	14.3% (4)	10.6%
T C C T	4.3% (3)	3.3% (2)	4.1% (4)	3.6% (1)	3.9%
C C T C	0	1.7% (1)	1% (1)	0	0.8%
C C C C	0	1.7% (1)	0	0	0.4%

**c.235delC-haplotypes (5-6-7-11)**	**Chinese** [7] (n=22)	**Japanese** [7] (n=12)	**Koreans** [7] (n=6)	**Altaians** [21] (n=8)	**Mongolians** [7] (n=4)	**Buryats** [This study] (n=2)	**Total** (n=54)
**G A C T**	**100% (22)**	**91.7% (11)**	**100% (6)**	**100% (8)**	50.0% (2)	**0**	**90.7%**
G A T T	0	8.3% (1)	0	0	0	0	1.9%
**G A C C**	0	0	0	0	50/0% (2)	**100.0% (2)**	**7.4%**

Common haplotypes are underlined and in bold; n – number of chromosomes.

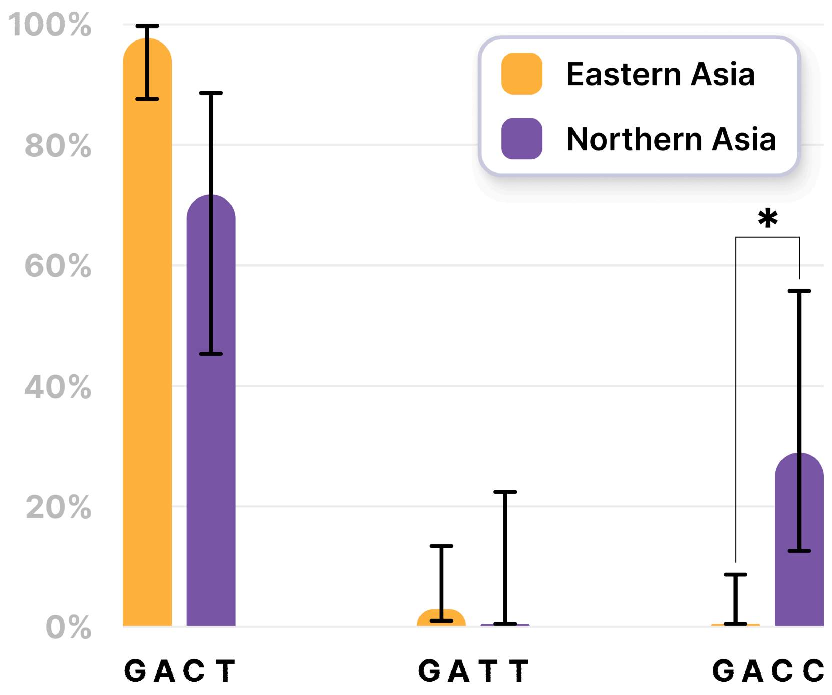

Figure S4. The c.235delC-haplotypes frequency in Eastern Asia and Northern Asia.
Eastern Asia (n=40): Chinese (n=22) [7], Japanese (n=12) [7], Koreans (n=6) [7];
Northern Asia (n=14): Altaians (n=8) [21], Mongolians (n=4) [7], Buryats (n=2) (this study), 95% Credible Interval, * - significant differences (*p*<0.05).
